# Functional outcome and athletic level after arthroscopic repair followed by triple pelvic osteotomy in patients with labral tears resulting from acetabular dysplasia

**DOI:** 10.1007/s00132-023-04399-x

**Published:** 2023-06-26

**Authors:** Ayham Jaber, Yannic Bangert, Katharina Gather, Sébastien Hagmann, Tobias Renkawitz, Alexander Barié

**Affiliations:** 1 Department of Orthopaedics, Heidelberg University Hospital, Schlierbacher Landstr. 200a, 69118 Heidelberg, Germany; 2 Center for Joint Surgery and Sport Injuries, Clinic St. Elisabeth Heidelberg, Max-Reger-Straße 5–7, 69121 Heidelberg, Germany

**Keywords:** Hip dysplasia, Pelvic osteotomy, Hip arthroscopy, Labral tear, Acetabulum, Hüftdysplasie, Beckenosteotomie, Hüftarthroskopie, Labrumschaden, Acetabulum

## Abstract

**Background:**

Patients with acetabular dysplasia are at a higher risk of developing symptomatic labral tears. Isolated treatments that address these pathologies are well established. Combined treatment with hip reorientation osteotomy using Bernese periacetabular osteotomy in addition to arthroscopic labral repair show good results. Studies that report the outcome in patients who received both arthroscopic labral repair and a triple pelvic osteotomy (TPO) are lacking. The aim of this study is to investigate the short to midterm functional outcome and activity level in these patients.

**Methods:**

This case series retrospectively included 8 patients (2 male, 6 female) with acetabular dysplasia (lateral center-edge angle [LCEA] ≤ 25°) and a labral tear on magnetic resonance arthrography (MRA). All patients underwent arthroscopic labral repair followed by TPO after an average period of 3 months (range 2–6). Average age at the time of surgery was 25 years (range 15–37). Patients were followed up and the following main parameters were assessed: LCEA, modified Harris hip score (mHSS), Tegner score, UCLA score, patient satisfaction on a scale of 1–4.

**Results:**

The mean follow-up was 19 months (range 15–25). The mean LCEA increased from 18° to 37° (p < 0.0001). The mHSS improved from a mean of 79 to 94 on final follow-up (*p* = 0.00123). The Tegner and UCLA scores had a median of 4 and 5, respectively. The mean LCEA increased from 18° to 37° (*p* < 0.0001). The mean patient satisfaction was 3.6.

**Conclusion:**

Patients with evidence of a labral tear resulting from acetabular dysplasia benefit from arthroscopic repair followed by a TPO. The literature still lacks evidence that labral repair and reorientation osteotomy produce superior outcome compared to osteotomy alone. Treatment should consider clinical presentation in addition to radiological findings with emphasis on MRA.

## Abstract (de)

**Hintergrund:**

Patienten mit Hüftgelenkdysplasie haben ein erhöhtes Risiko für die Entwicklung eines symptomatischen Labrumschadens. Spezifische Therapien dieser Pathologien sind bereits etabliert. Behandlungen, die eine Reorientierungsosteotomie der Hüfte mit einer periazetabulären Osteotomie nach Berner zusätzlich zu einer arthroskopischen Labrum-Rekonstruktion kombinieren, zeigen gute Ergebnisse. Aktuell exsistieren keine Studien über die Behandlung von Patienten mit einer arthroskopischen Labrum-Rekonstruktion kombiniert mit einer Triple-Osteotomie des Beckens (TPO). Ziel dieser Studie war, die kurz- bis mittelfristigen funktionellen Ergebnisse und das Aktivitätsniveau dieser Patienten zu untersuchen.

**Methoden:**

Diese retrospektive Fallserie umfasst 8 Patienten (2 Männer, 6 Frauen) mit einer Azetabulumdysplasie („lateral center-edge angle“ [LCEA] ≤ 25°) sowie eines Labrumschadens. Alle Patienten erhielten eine arthroskopische Rekonstruktion des Labrums, gefolgt von einer TPO. Diese erfolgte durchschnittlich nach 3 Monaten (Range 2–6). Das Durchschnittsalter zum Zeitpunkt der Operation betrug 25 Jahre (Range 14–36). Die Patienten wurden nachuntersucht und die folgenden Hauptparameter wurden erhoben: LCEA, modifizierter Harris-Hüft-Score (mHSS), Tegner-Score, UCLA-Score, Patientenzufriedenheit auf einer Skala von 1–4.

**Ergebnisse:**

Die durchschnittliche Nachbeobachtungszeit betrug 19 Monate (Range 15–25). Der mittlere LCEA stieg von 18° auf 37° (p < 0,0001). Der mHSS-Mittelwert verbesserte sich von 79 auf 94 bei der letzten Nachuntersuchung (*p* = 0,00123). Die Tegner- und UCLA-Scores hatten einen Mittelwert von 4 bzw. 5. Die mittlere Patientenzufriedenheit lag bei 3,6.

**Schlussfolgerung:**

Patienten mit einem nachweislichen Labrumriss infolge einer azetabulären Dysplasie profitieren von einer arthroskopischen Rekonstruktion gefolgt von einer TPO. Bisher gibt es in der Literatur keine Beweise, dass eine Labrum-Rekonstruktion mit Reorientierungsosteotomie bessere Ergebnisse liefern als die Osteotomie allein. Bei der Behandlung sollten vorrangig neben dem klinischen Erscheinungsbild insbesondere auch die radiologischen Befunde mittels Magnetresonanz-Arthrographie berücksichtigt werden.

**Graphic abstract:**

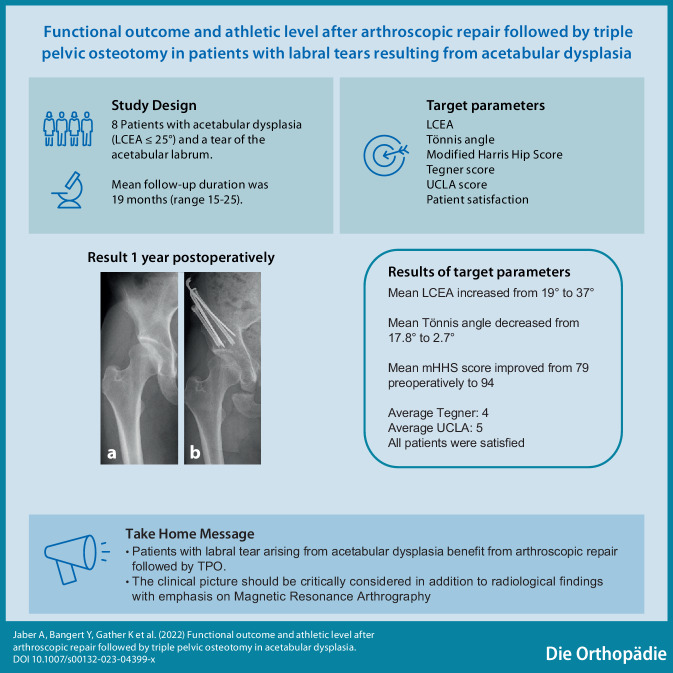

## Introduction

Acetabular dysplasia poses a proven risk of developing secondary osteoarthritis. The restoration of the anatomical and biomechanical state in the adolescent and adult populations delays or avoids the progression of joint destruction [1]. Several variants of pelvic osteotomy were established to achieve this goal. These mainly include Bernese periacetabular osteotomy (PAO), rotational acetabular osteotomy and triple pelvic osteotomy (TPO) procedures [2, 3]. Total hip replacement (THR) remains the last resort in end stage osteoarthritis with excellent results [4].

The overall rate of arthroscopic hip surgery is on the rise. In the USA, it increased 600% between 2006 and 2010 [5]. The most common hip pathologies include labral tears, lesions of the articular cartilage and femoroacetabular impingement [6].

Acetabular dysplasia is a recognized precursor for developing intra-articular pathologies [7]. The typical acetabular labrum in dysplastic hips is hypertrophied due to the lack of bony containment and the increased joint load delivered to the labrum. Acetabular dysplasia is one of several possible causes of labral tears [8]. Tearing of the labrum is likely the combination of overload because of the short and steep socket and microtrauma because of the reduced joint stability. As a result, the labrum of the hip joint undergoes hypertrophy and becomes instable. The eventual tearing of the labrum leads to a painful hip and even though a correction osteotomy leads to a better hip containment, the labral damage that resulted from the undercontainment is not dealt with. Pain could persist of if the labral damage is not addressed.

The recent medical literature examined the experience in treating these two related pathologies either separately or simultaneously. A recent review highlights this experience and concluded that arthroscopic repair is complimentary to correction osteotomy and should be considered in individual cases [9]. Most studies in the literature report results of PAO with, after or before arthroscopic repair [10]. These showed good results with a follow-up of up to 30 years [11]. The literature lacks results of TPO in this setting. The aim of this study is to investigate the functional outcome in these patients. It was hypothesized that hip arthroscopy followed by TPO leads to good results and a high activity level.

## Patients and methods

**Fig. 1:**
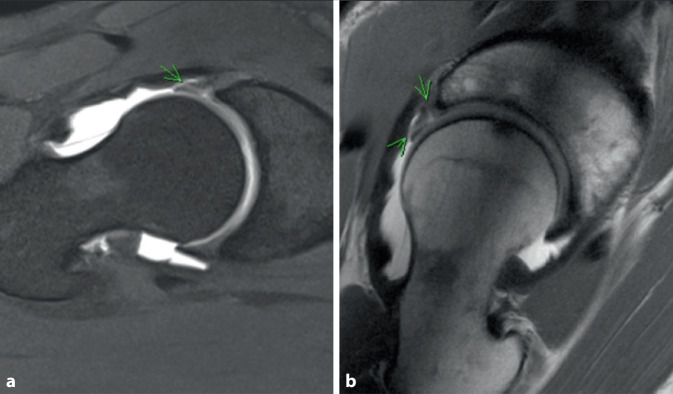
MRA of a 33-year-old female patient suffering from an anterior superior labral tear secondary to right-sided hip dysplasia indicated in axial (**a**) and sagittal (**b**) views

Between April 2016 and April 2018, 8 patients (2 male, 6 female) were consecutively included and retrospectively reviewed. All patients received arthroscopic repair (7 sutured and 1 partially resected) and then a TPO as a separate procedure between 2016 and 2018 for the diagnosis “acetabular dysplasia” as well as “labral tear”. Inclusion criteria included evidence of a labral tear on magnetic resonance arthrography (MRA) (Fig. 1), acetabular dysplasia with a lateral center-edge angle (LCEA) under 25°, radiographic preosteoarthritis or early osteoarthritis, skeletal maturity, improvement in joint congruency on anteroposterior (AP) radiograph with hip abduction, positive anterior hip impingement test on physical examination and absence of advanced cartilage damage seen during the hip arthroscopy.

Exclusion criteria included previous hip surgery at the effected side, advanced osteoarthritis, central neurological disorder and other underlying hip pathologies including those that could lead to labral damage such as relevant CAM and Pincer deformities. All surgeries took place in one university hospital and were performed by four senior surgeons.

The arthroscopic repair was carried out first in all patients. This was the standard order of the two procedures in the presence of the combined pathologies mentioned. The intraoperative positioning as well as the surgical technique was described by Dienst et al. [12].

The postoperative aftertreatment was similar in all patients. Following hip arthroscopy, patients were instructed to use crutches with partial weightbearing (20 kg) for a period of 2 weeks, followed by a gradual increase to reach full weightbearing within 2 weeks. The surgical technique of the TPO used was described by Tönnis et al. [3]. Kirschner wires in addition to cannulated screws were used according to the surgeons’ experience. The equipment for the osteosynthesis used in all patients is displayed in Table 1. Postoperatively, scores patients refrained from weight bearing for a period of 6 weeks followed by gradual weight bearing to reach full weight bearing within 2 weeks. Clinical, radiographic and functional evaluation was then performed at 6 weeks, 6 and 12 months and at 1‑year intervals thereafter following surgery. The duration of follow-up periods varied depending on the patients’ compliance with the given schedule.

**Table 1:** Patients listed in chronological order displaying results of preoperative and postoperative parameters

No.	Age (years)	Sex (male/female)	Interval between surgeries (months)	Labral suture or partial resection (PR)	Canulated screws/Kirschner wires used in TPO	LEA	LEA	Tönnis angle	Tönnis angle	Follow-up duration	mHHS (0–100)	mHHS (0–100)	Tegner (0–10)	UCLA (0–10)	Patient satisfaction (1–4)
						Preoperatively	Postoperatively	Preoperatively	Postoperatively	(months)	Preoperatively	Follow-up			
1	17	Female	6	1 Suture	0 CS/5 KW	18°	36°	11°	−3°	23	89	100	5	7	4
2	36	Female	3	3 Sutures	2 CS/3 KW	16°	42°	26°	5°	21	78	90	4	5	3
3	33	Male	2	3 Sutures	2 CS/4 KW	22°	38°	16°	5°	16	82	90	7	8	3
4	27	Male	4	2 Sutures	1 CS/4 KW	21°	32°	15°	9°	19	78	83	4	6	3
5	33	Female	3	2 Sutures	2 CS/3 KW	21°	33°	26°	1°	25	76	94	5	7	4
6	14	Female	2	3 Sutures	0 CS/5 KW	22°	47°	13°	−6°	21	81	100	5	7	4
7	16	Female	2	PR	2 CS/3 KW	15°	32°	19°	1°	19	69	100	6	7	4
8	25	Female	3	2 Sutures	3 CS/0 KW	17°	36°	17°	10°	15	79	94	5	7	4

The average period between the 2 surgeries was 3 months (SD 1.35 months, range 2–6 months). Average age at the time of surgery was 25 years (range 14–36). Patients were followed up and the following parameters were assessed: improvement in modified Harris hip score (mHHS) preoperatively and on final follow-up [13], Tegner score [14], UCLA score [15], patient satisfaction on a scale of 1–4, with 1 indicating “not satisfied” and 4 indicating “very satisfied”. Furthermore, anteroposterior (AP) radiographs were performed on follow-up to evaluate osseous healing and hip joint position postoperatively.

Data acquisition and analysis were performed in compliance with protocols approved by the ethics committee of the medical faculty of the Ruprecht-Karls-University Heidelberg (S-337/2018). The study was registered in the German Register of Clinical Studies and was conducted in accordance with the Declaration of Helsinki. All patients gave their written consent to participate in the study.

Descriptive statistics were used to report frequencies and means for the cohort and subgroups. A 2-tailed *t*-test was performed to compare preoperative and postoperative data. All statistics were performed using IBM SPSS Statistics (Version 27). *P*-value < 0.05 was considered statistically significant.

## Results

**Fig. 2:**
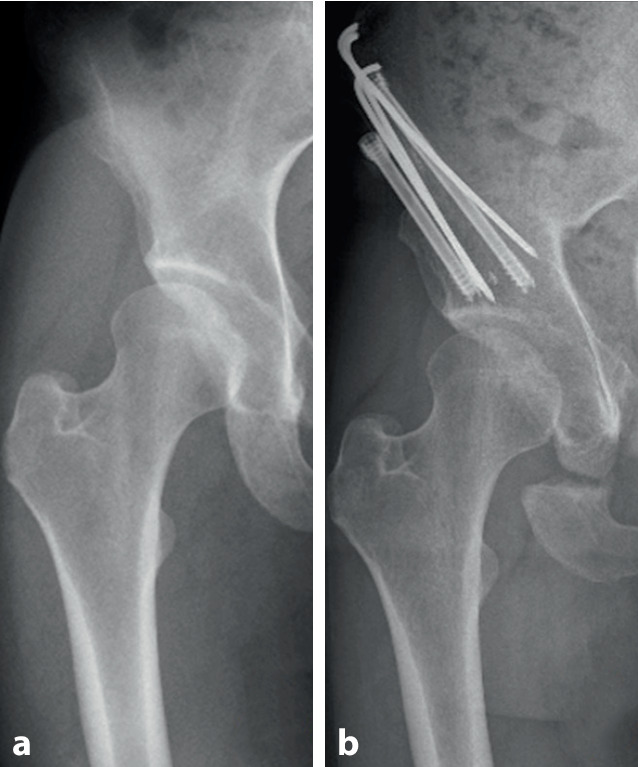
AP-radiographs of a 33-year-old female patient preoperatively (**a**) and 1 year postoperatively (**b**)

The mean follow-up duration was 19 months (range 15–25). No patients were lost to follow-up. The mean mHHS score improved from 79 (range 69–89) preoperatively to 94 (range 83–100) on final follow-up. This difference was statistically significant (*p* = 0.00123). Tegner and UCLA scored a median of 4 (range 4–7) and 5 (range 5–8), respectively. The mean LCEA increased from 19° to 37° (*p* < 0.0001). The mean Tönnis angle decreased from 17.8° (range 11–26°) to 2.7° (range −3–10°). The mean patient satisfaction was reported to be 3.6 (range 3–4). All patients were satisfied with the result on final follow-up. One minor complication in the form of sensory deficit of the lateral femoral cutaneous nerve occurred in 1 patient. Radiological imaging on follow-up showed full osseous healing of the ilium in all patients as seen in Fig. 2. Metal extraction was performed in 5 patients after complete osseous healing. The results are thoroughly presented in Table 1.

**Fig. 3:**
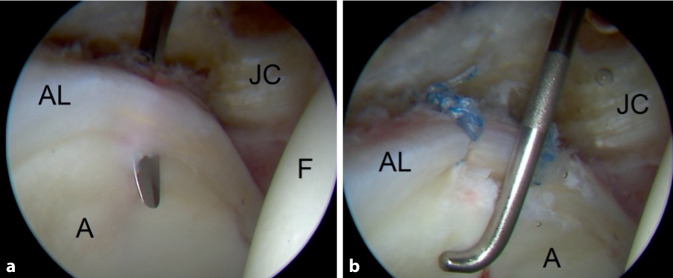
Intraoperative images of the hip arthroscopy in a 33-year-old female patient. **a** Full thickness labral tear of the superior labrum. **b** Results after repair using 2 Juggerknot anchors. *AL* acetabular labrum, *A* acetabulum, *F* femur, *JC* joint capsule

Regarding intraoperative findings of the arthroscopy, all patients showed a hypertrophied and instable labrum. No relevant cartilage degeneration was seen. There were no patients who did not receive a TPO because of the intraoperative finding of the arthroscopy. In most patients, a labral tear was identified and repaired using 1–3 Juggerknot Anchors (Biomet, Warsaw, IN, USA) (Fig. 3) and in the case of a complex tear or a labral degeneration, a partial resection was done. No bony resections were done.

## Discussion

This small case series reports good results after arthroscopic repair of the acetabular labrum and reorientation of the hip joint using TPO. In the medical literature, the Bernese PAO is referred to as the gold standard for treating hip dysplasia. It offers reproducible results with good long-term survivorship [16]. It is, however, technically demanding. The TPO described by Tönnis reported a good and reproducible clinical outcome [17]. It is therefore performed as standard in several institutes. Studies that reported results of TPO with relation to hip arthroscopy are lacking. The present study reports the first results in this setting. In the presented study, the treated hips had excellent clinical results on short-term follow-up. All patients were satisfied with the surgical interventions. The strengths of the present study include diversity of outcome measures which include several scores, as well as strict inclusion and exclusion criteria. The limitations mainly include the small number of patients. However, it is reasonable to report preliminary results of this 2‑stage treatment before it develops into standard practice. In the present study, all patients received the arthroscopic repair before the pelvic correction. It remains unestablished, whether the order of the two procedures has a significant effect on the results. The authors of the present study sought to treat the labral tear first which was diagnosed and localized on the MRA. This is technically easier to perform in the dysplastic hip. Moreover, a direct visualization of the hip joint is advantageous in order to exclude any patients with contraindications for the triple osteotomy, such as advanced cartilage damage to the joint. Additionally, the authors wanted to avoid traction positioning of the patient after freshly receiving a TPO.

The use of hip arthroscopy alone in the setting of symptomatic moderate to severe acetabular dysplasia remains controversial as it does not address the underlying structural deformity. Some studies demonstrated good outcomes, emphasizing that outcomes were more correlated with the type of intra-articular pathology, independent of the presence of dysplasia [18]. On the other hand, poor outcomes were reported in patients with dysplastic hips who received isolated hip arthroscopy, even suggesting that the surgery may accelerate the arthritic process [19]. Even if the resulting labral lesion is addressed, the etiology persists, and labral damage can recur. An additional advantage of hip arthroscopy is visualizing and accurately grading chondral damage of the hip joint in a minimally invasive manner, which may aid in subsequent planning of the course of treatment; however, there is insufficient evidence to conclude that arthroscopic characterization alone has any bearing on the eventual clinical outcomes after osseous reorientation procedures.

Combined hip arthroscopy or arthrotomy and PAO reported good results in patients with intra-articular hip pathology resulting from acetabular dysplasia. Nevertheless, matched studies failed to report a better outcome in patients who received both procedures simultaneously compared to just POA alone [20]. This led to many surgeons being skeptical about the benefit of the additional procedure. Thus, standard practice remains variable and depends on the surgeons’ experience and preference. Matta et al. [21] reported positive results after PAO alone and the authors changed from routinely performing an arthrotomy with a PAO to just the PAO alone as they felt that the arthrotomy with labral repair had minimal to no effect on the outcome [22]. Matheney et al. reported a series of 135 hips in which 61% underwent an additional arthrotomy at the time of the PAO. The presence of a labral tear was a predictor of a worse outcome on long-term follow-up [23]. In contrast, Peters et al. changed their surgical approach to routinely combine an arthrotomy with the PAO due to concerns regarding a potential impact of intra-articular pathology on the outcomes [24]. Kim et al. reported 43 consecutive hips that were treated by combined PAO and arthroscopy, with significant clinical improvement after a mean follow-up of 74 months, and therefore recommended that PAO with concomitant hip arthroscopy be considered in all patients. Their findings with respect to HHS were similar to the results we reported [21]. The choice between singular or combined 2‑stage treatment remains therefore controversial. It would be interesting, however, to observe these patients in the long-term and whether a significant delay in the progression of associated osteoarthritis exists.

In our institute, considering the period mentioned when the patients were operated on, a total of 49 TPOs were done. Only 8 (16%) received a prior arthroscopy for labral repair before the TPO. The authors of the present study recommend a 2-stage approach to be decided on an individual basis which should be mainly based on the clinical presentation and the findings of the MRA concerning intra-articular pathology. In our institute, MRA and arthroscopic labral repair are planned in patients who present with symptoms that involve labral pathology [25]. Differentiating the clinical manifestation is not always straightforward. Hip, groin and buttocks pain due to a labral tear is relatively unspecific. The localization is usually related to the location of the labral tear [26]. Many patients with labral tears describe mostly a dull pain with intermittent episodes of sharp pain that worsens with activity. The onset of symptoms was described as insidious in 61% of patients [27]. Walking, pivoting, prolonged sitting, and impact activities, such as running, often exacerbate the symptoms and 71% of patients also described nocturnal pain [27]. Mechanical symptoms, such as clicking, locking, and “giving way” symptoms have been reported to be present in more than half of the patients with a labral tear [28].

The most consistent physical examination finding in patients with acetabular labral tears is a positive anterior hip impingement test which involves passively moving the hip in flexion, adduction, and internal rotation [29]. In the presented patient collective, all patients had a positive anterior hip impingement test on physical examination.

## Conclusion

Patients with evidence of labral tearing arising from acetabular dysplasia tend to benefit from arthroscopic repair followed by TPO. There is no specified interval between the two operations so far. Further comparative research remains indispensable since there is still no evidence to suggest that arthroscopic repair and pelvic reorientation osteotomy result in a superior outcome compared to pelvic osteotomy alone. Current treatment should be tailored to patients’ individual clinical and radiological presentation especially considering MRA.

## Declarations

All procedures performed in studies involving human participants were in accordance with the ethical standards of the institutional ethics committee of the medical faculty of Ruprecht-Karls-University Heidelberg (S-337/2018) and with the Helsinki declaration of 1975, as revised in 2013. Informed consent was obtained from all participants included in the study.

## References

[1] Gala L, Clohisy JC, Beaulé PE (2016). Hip dysplasia in the young adult. J Bone Joint Surg Am.

[2] Vaquero-Picado A, González-Morán G, Garay EG, Moraleda L (2019). Developmental dysplasia of the hip: update of management. EFORT Open Rev.

[3] Tönnis D, Behrens K, Tscharani F (1981). A new technique of triple osteotomy for turning dysplastic acetabula in adolescents and adults (author’s transl). Z Orthop Ihre Grenzgeb.

[4] Learmonth ID, Young C, Rorabeck C (2007). The operation of the century: total hip replacement. Lancet.

[5] Sing DC, Feeley BT, Tay B, Vail TP, Zhang AL (2015). Age-related trends in hip arthroscopy: a large cross-sectional analysis. Arthroscopy.

[6] Ross JR, Larson CM, Bedi A (2017). Indications for hip arthroscopy. Sports Health.

[7] Fujii M, Nakashima Y, Jingushi S, Yamamoto T, Noguchi Y, Suenaga E, Iwamoto Y (2009). Intraarticular findings in symptomatic developmental dysplasia of the hip. J Pediatr Orthop.

[8] Hartig-Andreasen C, Søballe K, Troelsen A (2013). The role of the acetabular labrum in hip dysplasia. A literature overview. Acta Orthop.

[9] Adler KL, Giordano BD (2019). The utility of hip arthroscopy in the setting of acetabular dysplasia: a systematic review. Arthroscopy.

[10] Spiker AM, Gumersell KR, Sink EL, Kelly BT (2017). Modifications to the hip arthroscopy technique when performing combined hip Arthroscopy and Periacetabular osteotomy. Arthrosc Tech.

[11] Lerch TD, Steppacher SD, Liechti EF, Tannast M, Siebenrock KA (2017). One-third of hips after periacetabular osteotomy survive 30 years with good clinical results, no progression of arthritis, or conversion to THA. Clin Orthop Relat Res.

[12] Dienst M (2006). Hip arthroscopy. Technique for positioning and distraction. Orthopade.

[13] Kumar P, Sen R, Aggarwal S, Agarwal S, Rajnish RK (2019). Reliability of modified Harris hip score as a tool for outcome evaluation of total hip replacements in Indian population. J Clin Orthop Trauma.

[14] Tegner Y, Lysholm J (1985). Rating systems in the evaluation of knee ligament injuries. Clin Orthop Relat Res.

[15] Zahiri CA, Schmalzried TP, Szuszczewicz ES, Amstutz HC (1998). Assessing activity in joint replacement patients. J Arthroplasty.

[16] Kamath AF (2016). Bernese periacetabular osteotomy for hip dysplasia: surgical technique and indications. World J Orthop.

[17] van Hellemondt GG, Sonneveld H, Schreuder MH, Kooijman MA, de Kleuver M (2005). Triple osteotomy of the pelvis for acetabular dysplasia: results at a mean follow-up of 15 years. J Bone Joint Surg Br.

[18] Byrd JW, Jones KS (2003). Hip arthroscopy in the presence of dysplasia. Arthroscopy.

[19] Parvizi J, Bican O, Bender B, Mortazavi SM, Purtill JJ, Erickson J, Peters C (2009). Arthroscopy for labral tears in patients with developmental dysplasia of the hip: a cautionary note. J Arthroplasty.

[20] Thanacharoenpanich S, Boyle MJ, Murphy RF, Miller PE, Millis MB, Kim YJ, Yen YM (2018). Periacetabular osteotomy for developmental hip dysplasia with labral tears: is arthrotomy or arthroscopy required?. J Hip Preserv Surg.

[21] Kim KI, Cho YJ, Ramteke AA, Yoo MC (2011). Peri-acetabular rotational osteotomy with concomitant hip arthroscopy for treatment of hip dysplasia. J Bone Joint Surg Br.

[22] Matta JM, Stover MD, Siebenrock K (1999). Periacetabular osteotomy through the Smith-Petersen approach. Clin Orthop Relat Res.

[23] Matheney T, Kim YJ, Zurakowski D, Matero C, Millis M (2009). Intermediate to long-term results following the Bernese periacetabular osteotomy and predictors of clinical outcome. J Bone Joint Surg Am.

[24] Peters CL, Erickson JA, Hines JL (2006). Early results of the Bernese periacetabular osteotomy: the learning curve at an academic medical center. J Bone Joint Surg Am.

[25] Beckmann NA, Bangert Y, Putz C, Götze M, Renkawitz T, Hagmann S (2022) Behandlung der Hüftgelenkdysplasie beim jungen Erwachsenen [Treatment of hip dysplasia in young adults]. Orthopädie (Heidelb) 51(9):763–774. 10.1007/s00132-022-04281-2 (German) 10.1007/s00132-022-04281-2 35867116

[26] Hase T, Ueo T (1999). Acetabular labral tear: arthroscopic diagnosis and treatment. Arthroscopy.

[27] Groh MM, Herrera J (2009). A comprehensive review of hip labral tears. Curr Rev Musculoskelet Med.

[28] Farjo LA, Glick JM, Sampson TG (1999). Hip arthroscopy for acetabular labral tears. Arthroscopy.

[29] Burnett RS, Rocca DGJ, Prather H, Curry M, Maloney WJ, Clohisy JC (2006). Clinical presentation of patients with tears of the acetabular labrum. J Bone Joint Surg Am.

